# “We tell them it is the ‘fire age:’” gender, puberty education and adolescent boyhood in two Ethiopian contexts

**DOI:** 10.3389/frph.2026.1813598

**Published:** 2026-05-22

**Authors:** Sarah C. Blake, Allie Carney, Kahsay Sibhat Gebremedhin, Fikreselassie Getachew, Marni Sommer

**Affiliations:** 1 Department of Sociomedical Sciences, Columbia University Mailman School of Public Health, New York, NY, United States; 2 Grow & Know, Inc., New York, NY, United States; 3 Grow & Know, Inc., Addis Ababa, Ethiopia; 4 Ethiopian Public Health Institute, Addis Ababa, Ethiopia

**Keywords:** adolescent health, boys, Ethiopia, health education, masculinity, puberty, puberty education

## Abstract

**Introduction:**

As in much of sub-Saharan Africa, adolescent boys constitute a large and increasing share of Ethiopia's population. Masculinity norms, social, and structural factors intertwine to negatively impact boys' and men's health. What, where, and how boys learn about body changes during early adolescence is an important foundation for health over the life course. However, little evidence exists on boys' social and physical transitions in early adolescence.

**Methods:**

This exploratory qualitative and participatory study applied a social constructionist approach. We gathered insights on boys' learning and interpretation of body changes and their gendered implications. We collected data in two sites: one urban and one rural, representing distinct regions of Ethiopia. Methods included Participatory Activities with groups (*n* = 8) of adolescent boys (*n* = 120); Focus Group Discussions (*n* = 6) with primary school teachers (*n* = 32); and Key Informant Interviews (*n* = 15) with adults. Using reflexive thematic analysis, we used a multi-stage approach to code data, triangulate among data sources, and identify themes relevant to understandings of boys’ puberty and early adolescence.

**Results:**

We identified three themes related to boys' experiences of puberty and masculinity norms: 1) the “fire age” is central to male adolescence; 2) boys feel ambivalent about community masculinity norms; and 3) inadequate guidance leaves boys struggling to interpret body changes.

**Discussion:**

Findings suggested that boys were internalizing and struggling with normative definitions of adolescence and manhood, leaving them unprepared for puberty. A normative understanding held male adolescence as naturally volatile, with boys having limited ability to control their bodies. This fit with broader hegemonic masculinity norms prizing male physicality and justifying gender inequality through biological difference. Despite the centrality of puberty in shared understandings of male adolescence, boys had little guidance on interpreting or managing body changes. The study contributed to global research on the importance of understanding how and what boys learn in early adolescence and puberty. Puberty education should address Ethiopian boys' gendered experiences in tandem with scientifically accurate information.

## 1 Introduction

With more than 1.3 billion adolescents in the world, the factors that shape the transitions to adulthood are of critical importance (1). As in much of sub-Saharan Africa, adolescents account for a large and growing proportion of Ethiopia's population, More than 20% of Ethiopia's population of 135 million is between 10 and 19 years of age, and approximately 15 million are boys (1). Although, adolescent mortality is comparatively low and declining, preventable causes, such as injury, alcohol and drug abuse, and suicide persist as major risks (1–3). Further, adolescence is a pivotal “window of opportunity” for building health knowledge, promoting gender equitable attitudes and relationships, among other important factors in shaping health over the life course (1, 4–11). While evidence shows masculinity norms are critical to men's health across the life course, there is less attention to how boys learn about puberty and body changes in relation to broader understandings of what it means to successfully “become a man” (2, 6, 11–13).

Gender norms, or the assorted messages, rewards, and punishments that comprise the “unwritten rules” for behavior, appearance, relationships, and opportunities related to becoming a man or woman, sharpen in adolescence (3–5, 11). Masculinity norms intersect with broader social conditions, such as economic inequality, to shape boys' and men's health (12). While features of hegemonic masculinities vary by context, men often learn that power and social status come from qualities such as independence, dominance over both women and girls, and other men or boys, and sexual prowess (12). Public health research and interventions increasingly call for applying a gender lens to understanding and addressing boys' and men's distinct patterns of health behaviors and outcomes (13–15). This emerging body of research underscores how boys' and men's health risks are shaped by gendered dynamics and structural conditions rather than purely biological factors (2, 7, 11, 13, 14, 16). Despite the common use of male biological traits to explain risks, the meaning assigned to male physical features is underexplored in this research.

Early adolescence marks an important period for boys' encounters with gender norms. Across many contexts, including in Ethiopia, adults treat menarche and girls' changing appearance in puberty as markers of increased vulnerability, and caregivers often restrict their movements to shield them from male sexual attention (3, 6). By comparison, caregivers may allow boys more independence freedom of movement and access to opportunities (3, 17). Yet, evidence suggests this also may reflect underlying restrictive gender norms and practices. Boys face more violent discipline at school and home (16), suggesting that they, too, experience gendered risks. Such treatment can further enforce a norm that treats physical punishment as necessary and legitimate (16).

Boys' puberty is under-explored across global contexts (18). However, available evidence from contexts, including Kenya, Tanzania, and Cambodia suggests that such experiences are often marked by shame and silence (18–21). Boys in such studies have reported a lack of guidance in interpreting body changes from parents and schools (20–22). This has been documented to leave boys uneasy, anxious, confused and reliant on incomplete or inaccurate sources of information, such as peers or social media (19, 21). Further, boys may seek to demonstrate “manhood” by concealing feelings of uncertainty or vulnerability (20, 21). Evidence on related topics, such as families’ and schools' treatment of boys' sexual and reproductive health education, highlights complex sexual socialization processes. Across various contexts that like Ethiopia, emphasize sexual abstinence, boys may learn that sex is shameful and taboo, but also a source of male social status (23–25). This contradictory messaging may in turn, heighten anxieties and contribute to sexual and reproductive health risks, or justify violence against girls and women (19, 24, 26).

Ethiopian boys' ability to successfully transition to “become a man” in terms of achieving economic independence is a matter of substantial policy and public attention. Since 2004, when the government enacted the Youth Policy (17, 27), Ethiopia has passed policies and supported interventions focused on “adolescence” as a life stage, characterized by overlapping and interconnected education, health, and social needs (17, 28, 29). Although these efforts often highlight girl-specific aims, measures also address issues such as improving educational access or income earning opportunities for boys and young men (17, 29, 30). However, implementation has lagged due in part to ongoing social and economic instability (27, 31). While school attendance has increased, inadequate funding for teacher training, pay, and schools' built environments continue to constrain educational quality (17). Shortcomings in quality further contribute to ongoing struggles to ensure learners progress or complete school at intended ages (28, 30). This presents distinct challenges to delivering timely health education (32). Barriers to learning and educational progression, and a dearth of well-paying jobs for graduates also threaten boys' social transitions toward economic independence, potentially increasing unease and stress during this period (17, 31). What is more, it is unclear how concerns around boys' social, educational and economic prospects may relate to either early adolescence or preparation or experiences of body changes.

While there is a growing body of research on boys' experiences of puberty, there is still very limited evidence on how boys are learning about and navigating puberty and adolescence across low- and middle-income contexts, including Ethiopia. This study had the overarching objective of exploring boys’ perspectives and experiences of puberty. It sought insights on how gender norms and beliefs, formal, school-based puberty education, and informal learning are influencing Ethiopian boys' experiences of body, social, and emotional changes during their transitions to adulthood. It addressed two research questions:

1. What and how boys learn about and interpret body changes associated with puberty? and
2. How do body changes relate to broader social expectations for adolescent boys?

## 2 Methods and materials

We conducted a qualitative and participatory research study in one urban and one rural site in Ethiopia. The study used three methods: 1) Participatory Activities (PAs) with groups of adolescent boys ages 16–19 years old; 2) Focus Group Discussions (FGDs) with primary school teachers; and 3) Key Informant Interviews (KIIs) with adults with knowledge of boys' transitions through adolescence. Participatory methodologies can be useful for research with adolescents, including potentially sensitive, nuanced topics related to body maturation and new social pressures (4, 33). We purposively engaged older adolescents for their ability to reflect on their own transitions and provide recommendations to younger boys.

### 2.1 Research setting

We conducted research in two research sites representing distinct economic, ethnic, and linguistic contexts within Ethiopia. The rural site was a large town in the ethnically and linguistically diverse Gedeo Zone, located at the time of data collection in the Southern Nation and Nationalities People's Region (SNNPR) (34). Because no census has been completed since 2007, precise population estimates for both regions are not available (35). According to government projections, Gedeo Zone has a population of approximately 1.3 million people, with more than 75 percent residing in rural areas (36). The region's economy is dominated by agriculture and specifically coffee production, and successive environmental and economic crises drive poverty, food insecurity, and high rates of both school dropout and child labor (37). Population estimates for Axum, the urban research location range from between 40,000 to 100,000 (36, 38). Tourism dominates Axum's economy, reflecting its status as an important historical and religious site in Ethiopian Orthodox Christianity (37). It is ethnically and linguistically homogenous, with nearly all residents belonging to the Tigrinya ethnic group. Study activities in Tigray were completed before the 2020–2022 armed conflict in the region (39).

### 2.2 Sampling and recruitment

To capture insights from boys with a range of life experiences, we recruited both in- and out-of-school boys in each site. Personnel at government secondary schools identified school-going adolescents and staff in youth activity or sports centers recruited out-of-school participants. The research teams encouraged school partners to recruit adolescent participants who represented diversity present among boys in each context. These included boys with variable academic achievement and engagement in school; and household economic status. In the rural site, which was more demographically diverse, they also sought participants who reflected multiple religious, ethnic, and linguistic backgrounds.

For focus group discussions, we recruited primary school teachers working in grades 4–8, which roughly correspond to ages 11–15, because of their experiences teaching boys through early adolescence. Key informants were purposively recruited based on their positions and ability to share observations of boys in various community settings. This included diverse professional positions, ages, gender, and parenting or occupational experiences to gather a spectrum of perspectives on boys and their behavior, development and educational needs. Key informants included parents, health workers, religious leaders, and government and community officials, such as Kebele chiefs (ward leaders), youth leaders, and police officers responsible for youth affairs. The study team worked with local government and school officials to identify and recruit participants who fit common profiles in each site.

### 2.3 Data collection

Data collection began in late 2019, in in urban Tigray. However, it was interrupted due to the COVID-19 pandemic and subsequent civil conflict. Data collection resumed in the rural SNNPR site in late 2022. The data collection team included one American female researcher (AC) and two Ethiopian male research assistants (KS, MD), one from each region. Teams spent one month in each site. Research was conducted in Tigrinya and English in the urban site, while the team used Amharic, Gedeoiffa or English in the rural site. All research participants provided informed verbal consent in the language in which they felt most comfortable. To maintain confidentiality, teachers were not allowed in meeting spaces. Activity prompts were designed to reduce the likelihood of sharing potentially sensitive or embarrassing individual experiences in group settings. The research team reminded participants not to include their names on written materials. At the beginning of each group session, researchers reviewed how to contact local service providers with participants and encouraged them to access support if they experienced distress.

With participant consent, all KIIs and FGDs were audio recorded, and then translated and transcribed into English for analysis. Due to potential embarrassment that might arise out of discussing body changes and other puberty-related topics, and the challenges of recording activities with multiple breakout groups, the PA groups were not audio recorded. Each PA activity generated data in multiple formats: participant-written and generated data from group brainstorms, and notes recorded by the research assistants during the activities. The research team took extensive notes on group brainstorms, and collected written, participant-generated data from small groups and individual participants. After sessions, the study teams reviewed notes, and integrated all data into single documents to ensure completeness of data. For individual anonymous stories, boys were invited to write and turn in responses, which provided a distinct form of participant-generated data. Researchers gave out-of-school boys the option to privately narrate individual stories using the voice note app on the lead researcher's phone to ensure those with limited literacy could fully participate. For boys who audio recorded stories, researchers transcribed stories and deleted the recording immediately following transcription.

The three methods included:

*Key Informant Interview*s (KIIs): KIIs were conducted with adults (total *n* = 15; *n* = 7-8 per site) who regularly interact with adolescent boys, such as healthcare workers, parents, teachers, police officers, religious leaders, and policymakers. We used a semi-structured interview guide to explore adults' perspectives on boys' transitions into and through puberty and adolescence; community expectations for boys' transitions into manhood; and the suitability of school environments for supporting adolescent boys. Each interview lasted approximately 1.5 hour. To ensure confidentiality, interviews were held in a private room of the participant's choosing.

*Focus Group Discussions (FGDs)*: FGDs were conducted in primary schools (*n* = 6 groups, 3 groups per site; *n* = 6-8 teachers per group) in mixed groups of male and female teachers. Each FGD lasted approximately 1.5 hour. A semi-structured guide explored topics such as teachers' perspectives on the instruction schools provide on puberty-related topics; shifts in boys' academic and social behavior during early adolescence; how peer dynamics and school environment influence boys' experiences; and teachers' recommendations for how to support overall healthy transitions to adulthood.

*Participatory Activities (PA)*: PAs were conducted with groups (*n* = 8 groups; 4 per site; 12–20 boys per group) of in-school adolescent boys aged 16–19 (total *n* = 120). In each site, there were four groups: three groups in secondary school and one group of out-of-school boys. The secondary school groups were organized by grade level: Class 10, 11, or 12 (C10, C11 C12). Out-of-school groups included a mix of ages and educational attainment prior to dropout. Each group met three times for 1-2 hour. To maintain confidentiality, groups were held in private classrooms in schools or youth centers. The PAs included a mix of group and individual activities conducted over three sessions (see Table 1). Small and large-group brainstorm sessions allowed participants to reflect and share perspectives, while individual experiences were shared only in anonymous writing.

**Table 1:** Selected participatory activities.

Participatory activity	Description
Body change questions	Individual anonymously written questions on any body change topic participants wanted to learn more about.
Body change stories	Individual anonymous written stories about a body change, such as erections or “wet dreams” (nocturnal emissions).
Becoming a man in Ethiopia	Small and large group brainstorm on what it means to “become a man” in Ethiopian society, including “what is good” and “what is bad” about expectations for men.
Puberty curriculum	Small and large group brainstorm to generate recommendations for what content, and at what grade, puberty education content should be delivered

### 2.4 Data analysis

The study team used a constructionist approach to guide a multi-step reflexive thematic analysis (40, 41). First, the researchers reviewed transcripts to familiarize ourselves with the data. Then, two researchers conducted open coding. We used a mix of inductive and deductive approaches to code and analyze a sample of materials from each method to draft a codebook. The full research team reviewed the draft codebook, code definitions and discussed initial code applications. Three researchers revised the codebook and coded all data in Dedoose. Two researchers then completed a second round of focused coding to refine analysis and identify themes emerging from patterns in the data. Throughout, the coding team engaged in memo-writing and regular review meetings to refine code definitions, guide code application, and inform theme development. During this stage, researchers explored: consensus and debate among participants regarding definitions or expectations for boys' development and roles; transmission or challenges to social norms; and on boys' puberty guidance and education needs. The coding team then shared draft themes with the full research team for review.

## 3 Results

Three themes were identified, including: 1) “The fire age” is central to boys' adolescence; 2) Boys feel ambivalent about masculinity norms; and 3) Inadequate guidance leaves boys struggling to interpret body changes**.** There were minimal differences in findings between the two sites.

### 3.1 Theme 1: “the fire age” is central to boys' adolescence

Adolescent and adult participants referenced a shared, biological deterministic, set of normative expectations around boys’ adolescence. In this understanding, physical changes of puberty mark entry into a period of upheaval and risk for boys: evidenced by common use of the term “the fire age.” Adults and boys alike agreed that boys learned about these social expectations before they reached adolescence, internalizing messages that while physical appearance became more adult, they were also naturally prone to increased conflict and disruptive behavior. Adult participants further characterized the “fire age” as a potential burden for parents, who would face community judgement for perceived failures to manage boys' behaviors.

Many adults and adolescents endorsed the idea that the rapid physical changes of puberty either caused or indicated boys' entrance into a period defined by risk-taking. Most adults described boys' physical development as a marker of “natural” reckless behavior that posed a risk to both individual boys and to the broader social order. Getting taller, developing muscles or facial hair could, in their view, lead to behaviors such as sexual initiation; gambling; using alcohol, tobacco, or khat; stealing; and fighting. According to a rural police officer, when a boy reached adolescence:

*The family and community considers [that] when he becomes physically mature they know there can be some behavioral change. It can be like not obeying orders or respecting the rules. So this is known in the community … especially above age 15. It is common that young boys become unwilling to respect the rules and their families as well as in school.* (GED–police officer)

An urban priest described the relationship between physical maturation and reckless behavior as a product of boys’ frustration with feeling physically mature, but lacking adult standing or maturity:

*He gets physical changes like his voice, hair grows on his pubic area, in his armpits and on his face. The boys start thinking they have power and start to have fights with others … It is because of their own physical change. They are between childhood and adult behavior, so they struggle to find their way.* (AXU - Orthodox priest)

Adolescent participants often used the idea of “the fire age” and associated expectations as if they were neutral descriptions of boys' development and behaviors. Yet, adolescent and adult participants alike indicated that adults taught boys to anticipate and apply the idea before experiencing changes themselves. As one rural teacher explained:

*We tell them that they are now in the Fire Age … at this age, changes might be happening with your body, emotions, and behavior. Physically they become strong with their shoulders widening, and it might lead them to changing their behavior.* (GED - FGD - 2)

In a brainstorming activity about the features and timing of puberty, a group of rural boys shared that they learned from many sources to anticipate “excitability” rooted in biological changes. One boy explained that at age15, “everything is attractive to the boys … relationships, love, those things attract us. We learn about this from social media … [we] also learn about “fire age” from the community” (GED - C11). However, another member of the group suggested that boys anticipated and felt internal conflict between their newly emerging emotions and desires, and religious messages condemning nonmarital sex: “boys like having contact with girls - like love relationships - but most religions do not allow this [sex] before marriage, so we have to pray for (about) it.” This alluded to confusion and anxiety, as they saw no means to navigate unruly romantic and sexual feelings outside of the context of marriage. Similarly, an urban group (AXU - C11) identified adolescence as a period during which boys would naturally “get in conflict with others” and “become disobedient to your parents.”

Several key informants expressed concerns that boys’ adolescence could produce behaviors with enduring negative consequences. Some adults identified practices such as experimenting with alcohol as both risky and an understandable reflection of boys' natural curiosity. As an urban government official described, alcohol use heightened the threat of a loss of control that would eventually lead to school dropout:

*When they take alcohol, this leads them to fighting more with their friends and also stopping their education. If they do not stop education, it gets very weak after they start taking alcohol.* (AXU - Youth Affairs Coordinator)

A rural government official voiced a common concern among many adults, framing boys as naturally unable to control themselves, and prone to cascading harmful behaviors:

*After they become mature [in terms of] body and physical development, they force themselves to have early marriage … around age 18 … Sometimes it is based on their own interest and others, I perceive, it is [because] they cannot control their feelings … Sometimes maybe they become hopeless when they drop from school … they directly make a marriage* (GED - Deputy Mayor)

In this shared view, broader contextual conditions, such as poor educational quality, raised the threat that the anticipated natural volatility of adolescence would have lasting negative consequences. Specifically, the prospect that boys would leave school and seek to marry, threatened their ability to achieve respectable adult status.

In addition to the potential impact of the “fire age” on boys’ futures, many adults described it as creating an obligation for parents, seeming to reflect a linked parenting norm. In this general view, boys would not be able to control their own behavior, leaving parents with the moral responsibility to ensure that boys safely transitioned through this period. In a few cases, however, participants suggested that structural conditions, such as household poverty, explained families' inability to provide the support or guidance boys needed. However, for the most part, discussions focused on what participants perceived as failures in parents' character. A rural police officer attributed what he saw as boys' inherent susceptibility to school dropout to family morality:

*Those adolescents from good families, the families that have been following up on their boys, they have good ethics and they are learning their education and are more productive … But there are adolescents who are badly behaving, because their family leaves them without any control and follow up. They are not getting educated because of this.* (GED - Police officer)

A rural male nurse expressed a more nuanced view, describing parental skill in recognizing natural developmental status and refraining from harsh parenting as important defining features. He attributed struggles arising due to parents' inability to make sense of this developmental stage “[a] family that does not accept the change of behavior … they are not aware of puberty stage, so they do not understand why the boy does not obey the orders.” In this common view, parents' frustration with such behavior could then escalate to aggressive punishment of boys, serving to only increase conflict.

Several adults and adolescent participants characterized the “fire age” as becoming riskier due to boys' increasing access to mobile phones and mass media. One urban priest demonstrated a common view that social institutions were losing moral and disciplinary authority:

*Mostly the things happening for boys is because the family and church no longer have full control of the boys, and things are happening outside of the family. These things are like khat, cigarettes, hashish, and having love relationships. They see these things on social media and on TV and because of seeing these things and not being fully mature, they do these unwanted things. They also have sex with girls and get diseases.* (AXU - Priest)

This illustrated a broader shared belief that new technology was adding to risks, as it appealed to boys' natural curiosity and excitable nature in the “fire age.” This outside influence could, in turn, overwhelm parents' individual and collective skills to manage boys during a volatile period. Such claims also reflected a broader sense of anxiety among adults that social changes were lessening control over boys.

### 3.2 Theme 2: boys feel ambivalence about masculinity norms

Across the two sites, numerous adolescents demonstrated ambivalence about some, but not all, elements of a dominant, partially physically determined, idea of “becoming a man.” They understood that adult manhood was constituted, first, by physical differences with women. “Becoming a man” also required economic independence, marriage, and fatherhood. Boys tended to agree that male physical attributes, such as having a muscular appearance or being able to perform physical labor, constituted natural sources of power and authority over women and children. Some questioned the idea that biology dictated strict divides in household responsibilities. Adolescents discussed the intense community pressure to fulfill obligations to meet social benchmarks of completing school, becoming economically independent, and marriage: all steps necessary to establish their own households and become a respected member of the community. Although aspirational, they also expressed anxieties about being able to earn money and provide for others' needs and desires.

Boys across the two sites shared a common understanding of adult manhood that revolved around the role of “administering the family.” As one rural adolescent group described, this was rooted in what they understood to be men's natural responsibility to provide guidance and direction for women and children: “For example, like bees, if the bees have no queen, the other bees will have no direction. So the father is like that of the queen of the bees, he is a leader and a role model for his family” (GED - C11). The bee analogy suggested that the participant not only perceived the male head of household as a natural leader, but that women and children were “naturally weaker.”

While boys agreed that men should have the most authority in the household, they demonstrated varied views on whether divisions of labor were also natural or were socially imposed. One urban participant attributed diverging roles as a reflection of differences in physical strength, “A man has the work like a carpenter or a farmer, which are jobs for men. Women are supposed to make the food but not have physical activity because they have weak muscles” (AXU - C12).

Some groups engaged in substantial debate over rigid divisions of gender roles. In one rural group, a participant asserted that men were naturally suited to physical labor, “Culturally, the labor activities are tasks for men, because the men have the energy needed to perform the activities.” Another group member argued in contrast, “It is mostly men who do these jobs, but women can do them too.” (GED - C12). Though it was not always clear why adolescent participants questioned this division of roles, some specifically felt that it was acceptable for women to earn money to, as one said, “reduce the pressures on men” (GED - C11).

Along with potentially allowing women to take on more money earning responsibilities, several boys challenged norms that kept men from learning household chores, specifically cooking. A rural boy expressed a common view questioning the gendered meaning of cooking:

*They label men “female-ish” if they participate in kitchen activities, so this needs improvement. We want to learn how to make fast foods like eggs. If we tried to do this now … we might not be able to do it, because we do not have the experience, due to the time we spend doing labor activities, so we would not deliver the food. There is no difference between men and women biologically to do this work, so naturally, men and women should share the kitchen work.* (GED - C11)

Further ambivalence around existing norms appeared in several participatory group discussions about “becoming a man” that related to participants' visions of ideal marital relationships. Many adolescent participants spoke about the value of “respect” for wives, and equal decision-making. However, another member of the group rejected the idea of sharing any power with women, and, further, referenced a common idea that women's autonomy would reflect negatively on their husbands as he declared:

*It is not good if your wife makes more money than you, because she will not respect you if she has her own money … The wife should be under control of the man. For example, you need to control your wife's sexual emotions and satisfy her sexually.* (AXU - O)

Even where participants asserted that men had an obligation to serve women's sexual needs, they described women's sexual fidelity as a fundamental marker of “respect” for men. Several groups agreed that women's sexual fidelity in marriage was both more important than any obligation for men, and/or that infidelity justified violence. Many boys agreed that while violence against women was wrong, it was sometimes necessary. A response from a rural group was typical: “If the wife has another sexual partner, this also should be punished with beating because there is no respect” (GED - C11). In a few cases, however, participants challenged the idea that violence was ever justified, seeming to support a more equitable norm. A member of a second rural group argued that: “It is important for the wife to respect the husband, that is what she needs to do, but it is never justified to beat her” (GED - C1O).

### 3.3 Theme 3: inadequate guidance leaves boys struggling to interpret body changes

Across sites, and among both adults and adolescent participants, there was a consensus that boys were not receiving adequate information or guidance about puberty. While it was not always clear how much adults knew about school and/or family settings, they tended to agree that discussions were limited in both. Adults and adolescents alike observed that boys viewed outward changes, such as developing muscles or getting taller as natural and desirable. However, they lacked practical preparation for various changes to expect and manage, including developing pimples, or facial and body hair. Developmental features of sexual maturation, such as erections and the onset of nocturnal emissions, were a particular source of stress and anxiety for boys.

Boys' written body change stories demonstrated how little they had learned about puberty. Even in middle or late adolescence, major knowledge gaps persisted. In discussions of their earlier experiences of puberty and puberty education, some boys recalled learning to recognize certain non-sensitive body changes, such as developing facial hair, marked progress toward adulthood. However, the dominant message most boys recalled in relation to puberty was the admonition to “*focus on [their] education*,” and not think about body changes at all. The anonymous body change stories illustrated how such messages often led to confusion, embarrassment, and shame around sexual maturation. One story from the urban site illustrated a common combination of negative feelings, which led the participant to conceal what had happened from his parents:

*I was 14 years old. I felt confused because it was new to me. When it [the wet dream] happened, I didn't tell anyone because I was feeling shame. When I saw [the semen] after I woke up, I changed my pants and I slept afraid until daytime. Then I went to a bathroom to wash my body … when they [my parents] learn about this, I will feel shame. There is no one that advised me.* (AXU - C12)

This suggested that even though the participant had little context and no advance preparation, the sexual nature of the dream induced shame and fear that made it impossible to discuss. In the few cases where boys reported telling anyone about what happened, they recalled going to a peer-age relative or friend. The mixed reactions that followed highlighted the absence of reliable adult guidance for both the boys and those they told. As one story from the urban site described, such peer sharing sometimes served as a source of reassurance:

*I was playing with one girl outside of town during the day. At the same time that night,* *I had a dream playing with her and I got a wet dream. At this time, I woke up and I said, “What happened to me?” and I went to change my pants, and I told my friend this thing happened to me, and he said, “Me too!” [so we started] to think this is normal.* (AXU - C10)

However, peers often proved to be unhelpful. Several participants described friends demonstrating negative or unsupportive reactions. One rural boy described his frustration with his friends' inadequate response:

*At the age of 16, while I was in bed at night, I was dreaming and then when I woke up in the morning, I saw my trousers were wet and I told this to my friends. After I told them, I became angry at myself, “Why did I tell this to them?” Because they didn't tell me anything about [it happening to] themselves.* (GED - C11)

This story reflected the participant's discomfort and confusion in the moment, and the failure to find reassurance. It further revealed uncertainty and anxiety about how to handle new sexual feelings. The same participant went on to write about how, he and his peers considered taking drastic measures to marry and leave school, only later gaining a more measured understanding that sexual feelings were natural and could be managed without having sex:

*After some time, they started to talk with me, “shall we learn, or shall we get a marriage?” … together, we prefer to learn and continue our education. I am proud of my decision that I decided to continue my education. Now at the night, most of the time, I do not face those kind of things, but sometimes, I have experienced the wet dreams, but it is normal for me, nothing new* (GED - C11)

It was not clear how long or seriously the participant considered the choice between school, and socially desirable transition to adulthood, versus marriage, the only acceptable outlet for his sexual feelings. However, the confusion and distress about sexuality and sexual expression was common. Along with narratives, the anonymous puberty questions that adolescent participants submitted also highlighted how many of them felt dismay and ongoing confusion about the changes happening as a part of puberty and adolescence. Wishes for “a pill” or other means to eliminate erections or nocturnal emissions until they were married were common, and often linked with questions such as, “How can we eliminate wet dreams? Give your advice.” (AXU - C12). Similarly, a participant from the rural site asked, “Why is it when boys look at beautiful girls, his penis becomes erect? Why does this happen and how can you control it?” (GED - C11). Given that the research was conducted with older adolescents, the ongoing bewilderment that boys expressed suggested that they still lacked critical knowledge about both physiological functions and their significance in relation to norms demanding abstinence until marriage.

Several primary school teachers and key informants indicated that neither families nor schools were providing the kind of supportive guidance that boys needed to accurately interpret sexual maturation. This appeared largely driven by adult discomfort. As one urban mother (AXU) declared, when it came to matters of sexual development, “we push those conversations down*.”* A rural Kebele (ward) chief placed responsibility on fathers, seeming to question parenting norms that he saw as impeding relationships with their sons:

*Boys don't talk to their fathers about their body changes because they [fathers] try to manage their boys by force. So the boys do not have a good relationship with them … Also, the fathers are always away from home, late into the night. So practically, it is hard for boys to ask them questions because they are not around.* (GED – Kabele chief)

While some adult participants were unfamiliar with school puberty education, teachers, the group who were most knowledgeable, saw schools as failing to provide timely and thorough puberty education. An urban teacher expressed a common view of the inadequacies in puberty education for boys: “They learn deep information on reproductive health in grade 8, but they need to be learning earlier, like in grade 6.” (AXU - FGD - 1).

Adult participants involved in education and health provision linked the lack of puberty education to harmful behavior among boys. According to a primary school teacher, boys who were unprepared for puberty would not ask for advice or guidance. In the teachers' view, this lack of preparation led to boys harassing girls: “the boys do not come to the teachers for advice, but we observe them in class … harassing the girls and bullying them, so we give them advice when we see that happening” (GED - FGD - 1. While it was not clear what “advice” entailed, the group agreed that if boys understood more about what puberty entailed, they might be able to self-regulate behaviors and allow girls to learn in peace. A nurse further called for schools to provide puberty education as part of a broader set of interventions to offset sexual and reproductive health risks:

*They need to get enough information at puberty about their bodies and gender equality. There should be centers in the school for them to learn, like youth friendly services … There are so many STIs [sexually transmitted infections] because there is no information for the lower grades. They need this information [about puberty]* (AXU - nurse, male)

Like adults, boys in both sites agreed that schools should provide more puberty education content, beginning in earlier grades. A group of urban boys reflected on their lack of education: “When we were in primary school, we did not learn about any of this [information about body changes], so students should learn that it is natural and an experience that everyone goes through” (AXU - C11). A group in the rural setting agreed that “in the textbook from grade 7 and 8, the puberty issues are incomplete.” (GED - C12). According to the boys, this information was necessary because of how it made boys feel. An urban group expressed common desire to avert “shock” and fear due to a lack of preparation:

*In grade 8, boys experience wet dreams, voice changing, and their shoulders broadening, so they need to learn these things in grade 5 to not get shocked … If boys have wet dreams, but they have not received information, they think they are sick.* (AXU - C12)

Some boys also described learning that they could control how they responded to sexual feelings. As one urban group suggested, in grade 6, “[boys] should know they do not have to have sex if they have these feelings and it is not advisable to do so at that age” (AXU - C10). These recommendations further suggested that participants generally endorsed a normative expectation to delay sex until marriage, but that they wanted guidance and support to fulfill this expectation.

Teachers across sites and groups supported expanding school-based puberty education for boys. Several asserted that primary school teachers like themselves could deliver content that untrained adults would find uncomfortable. According to a rural group, “The teachers have training in the physical and emotional changes, so they are able to teach.” (FGD - GED - 2). An urban teacher echoed this sentiment: “I am a biology teacher, so when I teach, it is very normal for me, but some students they laugh at first and then it becomes ok” (FGD - AXU-1). Finally, several teachers in both sites identified existing girl-focused approaches as models for programming with boys. One urban teacher explained: “For girls, they get advice and education about their menstruation and how to make their pads” (FGD - AXU-2). While it was not clear what specific content or guidance such programs provided, nor what kind of detail teachers envisioned providing for boys, there was a broad consensus that expanding such programs to reach boys was both feasible and necessary.

## 4 Discussion

This study explored boys' transitions through puberty and adolescence in two regions in Ethiopia: an urban site in the Tigray and a rural site in SNNPR. Through adults, boys learned to anticipate observable body changes, such as developing muscles, which served as markers of entry into a period of upheaval and risk. Findings highlighted how early adolescent boys were navigating internally contradictory masculinity norms: alternately internalizing, embracing, and questioning what they were learning about the meanings, entitlements, and obligations for adolescent and adult manhood. As boys expressed ambivalence about rigid adult masculinity norms and roles, they retained a belief that the ideal of male status as a “family administrator” was justified by biological difference. Specifically, they endorsed the idea that men's muscularity and physical labor conferred authority. Despite the clear salience of male physicality to adolescent and adult masculinity, silence and confusion surrounded boys' experiences of pubertal body changes.

Discussions of “the fire age” and its components underscored the importance of social norms and practices in boys' adolescence in Ethiopia. Similar to masculinity norms in other contexts, the concept rested on assumptions that male power and risk-taking were both natural and rooted in biological features differentiating them from younger boys and women (2, 12, 15). However, it was clear that adults were transmitting the concept through direct discussions with boys. Overall, this added to the limited available evidence on how communities in Ethiopia define adolescent boyhood. As in other contexts (6,42) volatility and a lack of control is expected, alternately tolerated or punished harshly (12, 15). Nonetheless, because the term was often used in passing, there was substantial ambiguity in terms of how the concept was transmitted, and the bounds of acceptable behaviors among boys it defined.

Our findings added nuance to existing evidence on masculinity and boys' adolescence in Ethiopia. For example, a study on adolescent and adult views on gender-based violence in schools found that teachers would invoke the concept of “the fire age” (26, 42) to excuse destructive and sexually aggressive behavior among boys. We observed a related expectation that boys lacked agency over their actions. However, for boys, learning about “the fire age” was not necessarily a reassurance or uninhibited permission to engage in reckless behaviors. Instead, the idea that they could not control behaviors appeared as a source of stress and anxiety, as they feared failing to complete school or achieve financial independence: critical markers of achieving manhood (43). However, further research is needed to explore how boys may internalize or question the idea of the “fire age” in relation to understandings of their own agency during this transitional period.

Adults' discussions of “the fire age” further illuminated potential distinct parenting norms and obligations. Specifically, adults suggested that observing boys' behavior would illustrate whether caregivers had the skills to conduct the right amount of “follow-up:” neither too permissive, nor too harsh of treatment. This suggested that along with common practices in Ethiopia and elsewhere that treat adolescent girls' sexual modesty as a reflection of family character (3, 44), parents also may face scrutiny for boys' behaviors. However, this did not suggest parents were expected to maintain control over boys movement to the extent that they would be with girls (6). Given this context, the rejection of harsh discipline was notable, as evidence suggests boys may bear the brunt of violent discipline because adults believe it is the only way to manage boys during a difficult developmental period (16). With “positive parenting” emerging as intervention approach in Ethiopia and other low resource contexts, future research and interventions should continue exploring gendered parenting norms as they apply to adolescent boys (45).

Among the risks referenced in relation to the “fire age,” was a concern of boys entering “early” marriage. “Early” marriage was clearly understood as a problem where boys failed to reach socially sanctioned transitions to manhood: completing secondary school, assuming a viable livelihood while remaining under the authority of their father, before marrying in their 20s and forming a household (3, 17, 46). This concern evoked, first, broader normative concerns about boys' perceived failures to achieve “manhood” in a context of poor quality education and limited economic opportunities (17). It also echoed the idea of “child” marriage, or marriage under age 18, which, in Ethiopia, typically involves family-sanctioned age disparate relationships between adult men and adolescent girls (47–49). Such marriages were considered “early” because young men have achieved economic independence, rather than reaching an age where they would be able to provide meaningful consent (43, 46, 48). Child marriages carry negative implications for girls' safety and health, due in part to power disparities between girls and much older husbands (47). There is very limited evidence on the potential social, health, and safety consequences of socially unsanctioned marriages between peers.

Similar to findings from other global contexts, many adults expressed anxieties that changing media and technology were threatening family and institutional authority and ability to provide guidance to boys (4, 20). Across contexts, it is common for adults to be preoccupied with potential threats to their authority over adolescents (20). At the same time, this highlighted a distinct documented evidence on the distinct negative role of mobile phones and social media on adolescent health and well-being for both boys and girls (14). Given that boys typically have greater access to phones than girls, understanding how boys engage with social media content may be particularly important (50, 51). There is a need for more in-depth research on how increasing access to mobile phones may affect health adolescent health across global contexts (52).

While adolescent participants endorsed the idea that muscularity justified adult men's authority to “administer the family” (48, 53), a fundamental component of hegemonic masculinity, they also debated the limits of such biologically determined gender roles. Every group debated absolute ideas of male authority. Several challenged biological justifications for women should be allowed to earn money, and men to learn to cook. This highlighted a potential opening to address gender inequitable norms, particularly where they contributed to gender role strain on men (15, 54). Notably, recent surveys have suggested that adult men may be adopting somewhat more gender equitable attitudes than previous generations, particularly in relation to household divisions of labor (55).

Beyond the division of gendered household roles, boys also appeared somewhat divided around deeper questions related to male dominance. There was general, but not unilateral, acceptance of the idea that violence against women was justified as a response to infidelity, and necessary to maintain “respect” for husbands (56). The latter finding was consistent with previously documented norms across Ethiopia justifying violence against women (27, 57). And, high levels of gender-based violence in Ethiopia constitute a long-standing matter of public concern, even as it may be normalized and tolerated in practice (26, 42, 47). Addressing the gender inequitable norms underpinning violence has been a priority for policy and interventions focusing on adolescent girls and older boys and men in Ethiopia (47, 57). Intervening in early adolescence may present a strategic opportunity to reach boys during a period where they are perhaps particularly sensitive to messages and models of masculine roles and behaviors (56).

The silence around body changes related to sexual maturation and boys' resulting shame and confusion represented a notable, if unsurprising, finding with important potential implications for health in adulthood. Gaps in supportive guidance from schools and family members are common across studies of boys’ experiences of puberty across many contexts, including in East Africa (19, 21, 58). For instance, studies from Kenya and Tanzania documented how fathers and sons maintained silences around body changes related to sexuality, leaving boys with both short-term fears that natural changes such as erections and wet dreams constitute a “disease” (20, 21). This finding added to broader evidence across contexts demonstrating caregiver anxieties related to discussing matters related to sex and sexuality with their children, particularly boys (25, 59, 60).

In contexts where sexuality norms and socialization emphasize shame and risk, these messages can lead young children to internalize the message that anything adjacent to sex is a source of shame, often contributing to negative reproductive health outcomes (24, 58, 61). Our findings clearly aligned with this point, as boys appeared to struggle to differentiate normal sexual maturation from prohibited sexual conduct. The common message to “focus on education” and not think about sex seemingly extended to body changes themselves, reinforcing boys' existing anxieties about confusing feelings and physical development. In Ethiopia, where schooling quality so often fails to deliver on the promise of supporting socially desired transitions to adulthood, such messages may be particularly discouraging (43, 46). However, further research is needed to explore dynamics related to broader norms and material conditions.

Findings on the shortcomings in puberty education largely aligned with the limited research on puberty education in Ethiopia. Past studies have documented insufficient in puberty education content for girls (32, 53). However, several teachers suggested that girls in their schools were receiving comparatively more content than boys. Although it was not clear what puberty education content girls might be receiving, there was a consensus that boys' distinct gendered learning needs may be particularly under-served (18). Adults agreed that schools were an appropriate venue improving puberty education for boys because it would complement existing efforts to improve guidance for girls. Given that there is exceedingly little evidence on effective approaches to addressing boys' puberty topics in Ethiopia or similar under-resourced school contexts (62), there is a clear need to design and test interventions appropriate for this “window of opportunity.” Teachers' willingness to deliver this content constituted a potential opportunity that could emerge if the well-established need to improve Ethiopia's education system (30) were addressed.

Overall, in contrast to the discussion of the “fire age” and boys’ experiences of their bodies as a source of emotional and social disruption, they received only generic reproductive biology content at school. This findings similar to other contexts (20, 21, 22), and underscored the importance of addressing puberty education as a gendered intervention, rather than a matter of factual knowledge alone. Continuing to refine research and interventions that address the meanings assigned to body changes in different contexts is important to fitting content to the lived experiences of puberty and adolescence. This should, fit within ongoing efforts to strengthen health education and gender transformative interventions across contexts (18).

### 4.1 Limitations

This study had a few limitations to note. Data collection was interrupted due to the COVID-19 pandemic and civil unrest, leading to a two year gap between data collection in the two sites. While this lag and the ensuing social instability might impact broader social conditions, there were minimal differences in responses. This may have reflected the retrospective nature of much of the project: adolescent participants were asked to remark on events in the past, while adults were invited to share broad perspectives on boys over time. Future studies may provide more in-depth insights on boys and their contexts by eliciting more reflections on social-political events or access to technologies relevant to boys, such as mobile phones. In addition, there was some potential for social desirability bias. It is possible that participants believed researchers had particular allegiances on a given topic, such as, for example, gender-based violence, or sexual health. To mitigate this risk, the study employed open-ended prompts and encouraged discussion and debate among participants in group activities. There was some initial confusion in the Tigray adolescent groups about the definitions of the local terms used for “puberty” and “adolescence.” Similarly, the study was limited to two distinct locations within Ethiopia: a large country with more than 80 ethnic groups and diverse geographic, economic, and social conditions, and, potentially, normative understandings and practices around boys' puberty and adolescent masculinity. Future studies should explore how various communities construct meanings around male adolescence, and how concepts such as “the fire age” may be used in regulating boyhood.

## 5 Conclusion

Our study explored how boys experience and interpret puberty and adolescent masculinity in a rural and an urban site, representing distinct regions within Ethiopia. Findings suggested that both puberty overall and distinct body changes carry anxiety for boys that remains largely unaddressed in school and home settings. The physiological process of puberty was infused with gendered meanings that boys internalized, even as they lacked practical guidance on puberty-related body changes. Future research should collect data on boys' puberty and puberty education experiences in a broader range of contexts across Ethiopia. It should also engage younger boys in order to better document the contemporaneous experiences of puberty and adolescence. Interventions should continue to expand health literacy among boys and create guidance that is age-appropriate and accessible to boys as they mature. Gender transformative interventions should tackle assumptions about boys' ability to control their own bodies and reject violence against women. Overall, efforts focusing on early adolescence should be linked with broader investments in Ethiopia's education and health infrastructure.

## Data Availability

The datasets presented in this article are not readily available because the IRBs did not approve release of the qualitative data collected for this study. Requests to access the datasets should be directed to Marni Sommer, ms2778@cumc.columbia.edu.
